# From Skeletal Muscle to Myocardium: Molecular Mechanisms of Exercise-Induced Irisin Regulation of Cardiac Fibrosis

**DOI:** 10.3390/ijms26083550

**Published:** 2025-04-10

**Authors:** Zhao Wang, Lin Li, Meng Yang, Biao Li, Siyuan Hu

**Affiliations:** 1 School of Traditional Chinese Medicine, Hunan University of Chinese Medicine, Changsha 410208, China; wangzhao@stu.hnucm.edu.cn (Z.W.); lilin@hnucm.edu.cn (L.L.); 20202064@stu.hnucm.edu.cn (M.Y.); 2 School of Physical Science, Hefei Normal University, Hefei 230061, China; 3 School of Sports & Arts, Hunan University of Chinese Medicine, Changsha 410208, China

**Keywords:** Irisin, exercise, cardiac fibrosis, cardioprotection

## Abstract

This study systematically elucidates the regulatory mechanisms and potential therapeutic value of the exercise-induced hormone Irisin in the pathological progression of cardiac fibrosis. Through comprehensive analysis and multidimensional data integration, we constructed a complete regulatory network of Irisin within the cardiovascular system, spanning its secretion, signal transduction, and precise regulatory control. Our findings demonstrate that exercise intervention significantly elevates circulating Irisin levels via the skeletal muscle–peroxisome proliferator-activated receptor gamma coactivator 1-alpha (PGC-1α)–fibronectin type III domain-containing protein 5 (FNDC5) signaling axis. Irisin establishes a multidimensional molecular barrier against cardiac fibrosis by targeting Sirtuin 1 (Sirt1) activation, inhibiting the transforming growth factor-beta (TGF-β)/Smad3 signaling pathway, and modulating the transcriptional activity of the mitochondrial biogenesis core factors PGC-1α and nuclear respiratory factor 1 (NRF-1). Moreover, the dual regulatory mechanism of the exercise–skeletal muscle–heart axis not only effectively suppresses the aberrant activation of cardiac fibroblasts but also significantly reduces collagen deposition, oxidative stress, and inflammatory infiltration by restoring mitochondrial dynamics balance. Taken together, this study reveals a novel exercise-mediated cardioprotective mechanism at the molecular interaction network level, thereby providing a theoretical basis for the development of non-pharmacological bio-intervention strategies targeting the Irisin signaling pathway and laying a translational foundation for precise exercise prescriptions in cardiovascular diseases.

## 1. Introduction

Cardiovascular disease (CVD) remains a leading cause of mortality and a major contributor to comorbidities worldwide [1]. Cardiac fibrosis, a central pathological mechanism underlying CVD progression, is ubiquitously observed in myocardial infarction, heart failure, and coronary artery disease [2,3,4]. Cardiac fibrosis not only impairs the structure and function of the myocardium, but may also increase myocardial stiffness, reduce compliance, and trigger severe complications such as arrhythmias. Ultimately, it can lead to irreversible cardiac decompensation, severely affecting patients’ quality of life and survival expectancy [5]. Consequently, the prevention and treatment of cardiac fibrosis have emerged as critical research priorities in the field of CVD. Recent advancements in disease intervention research have revealed that exercise training exerts potent anti-fibrotic effects in cardiac tissue [6,7,8]. Irisin, a novel exercise-induced myokine, has been identified as a key mediator of exercise-derived cardioprotective benefits [9]. Mechanistic studies demonstrate that Irisin modulates multiple signaling pathways involved in fibrogenesis, including the transforming growth factor-beta (TGF-β)/Smad signaling cascade, Sirtuin 1 (Sirt1)-mediated deacetylation pathways, and the peroxisome proliferator-activated receptor gamma coactivator 1-alpha (PGC-1α)/nuclear respiratory factor 1 (NRF-1) regulatory axis. These molecular interactions collectively regulate fibrotic remodeling, inflammatory responses, oxidative stress homeostasis, and mitochondrial bioenergetics [10,11,12]. However, how Irisin is produced in response to exercise and through which pathways it exerts its effects remains incompletely understood. Therefore, this study aims to systematically elucidate the molecular mechanisms underlying the exercise-induced fibronectin type III domain-containing protein 5 (FNDC5)/Irisin biosynthesis pathway, reveal its targeted transport characteristics from the circulation to the heart, and investigate the pivotal mechanisms by which Irisin modulates cardiac fibrosis and its potential clinical applications. This research provides new theoretical support and therapeutic insights for the prevention and treatment of cardiovascular diseases.

## 2. Current Status of the Treatment of Cardiac Fibrosis

**Figure 1:**
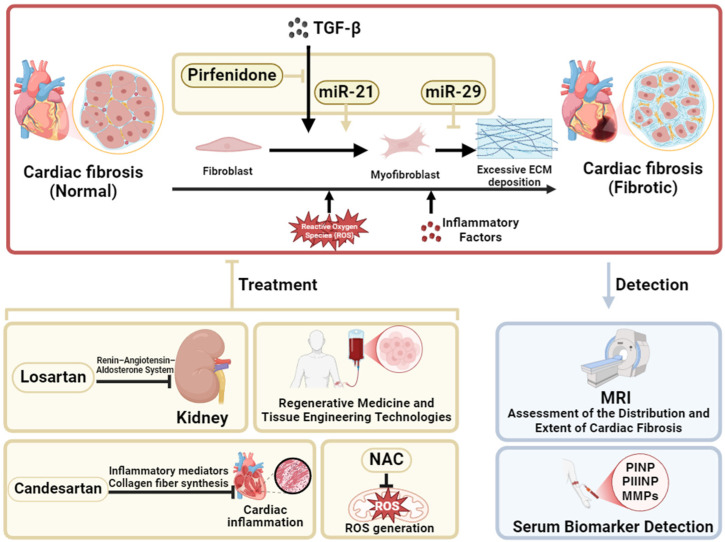
Mechanisms and Current Treatments of Cardiac Fibrosis. This figure outlines the mechanisms, detection methods, and treatment strategies for cardiac fibrosis. miR-21 promotes fibrosis progression by regulating the transforming growth factor-beta (TGF-β) signaling pathway, while miR-29 regulates the metabolic balance of the extracellular matrix (ECM). Oxidative stress and inflammatory factors further exacerbate pathological changes. Magnetic Resonance Imaging (MRI) and serum biomarkers can be used to assess fibrosis. Biomarkers include procollagen type I N-terminal propeptide (PINP), procollagen type II N-terminal propeptide (PIINP), and matrix metalloproteinases (MMPs). Drugs such as Losartan, N-acetylcysteine (NAC), and regenerative medicine techniques provide potential therapeutic options. This figure was created in BioRender. Wang, Z. (2025). https://BioRender.com/n80e582, accessed on 7 April 2025.

Cardiac fibrosis is primarily characterized by excessive deposition of collagen fibers in the myocardial interstitium [13]. This process is chiefly driven by the abnormal activation of fibroblasts and their subsequent differentiation into myofibroblasts. Upon stimulation, fibroblasts transform into myofibroblasts, which possess a markedly enhanced capacity for collagen secretion, leading to pathological accumulation of the extracellular matrix (ECM) [14]. Advances in molecular biology have deepened our understanding of the regulatory mechanisms underlying cardiac fibrosis. Among these, TGF-β is regarded as a key driver of fibrogenesis and is often referred to as the “fibrotic switch”. TGF-β activates downstream effectors associated with fibrosis via both Smad-dependent and Smad-independent signaling pathways, thereby promoting fibroblast activation, ECM deposition, and myofibroblast differentiation, which together accelerate the onset and progression of fibrosis [15,16]. Consequently, the TGF-β signaling pathway has emerged as an important target for antifibrotic therapies, with TGF-β inhibitors such as pirfenidone commonly used in clinical practice (Figure 1).

In reality, cardiac fibrosis is regulated by multiple molecular mechanisms, both directly and indirectly, necessitating different therapeutic agents to target various pathogenic pathways. For example, angiotensin-converting enzyme inhibitors, angiotensin II receptor blockers (ARBs), and aldosterone antagonists effectively reduce fibrosis by modulating the renin–angiotensin–aldosterone system (RAAS). Among these, losartan, as an ARB, not only mitigates the potent profibrotic effects of angiotensin II (Ang II) by blocking its receptor but is also found to downregulate TGF-β levels, thereby alleviating the progression of cardiac fibrosis [17,18]. Meanwhile, inflammatory responses play a significant role in cardiac fibrosis, prompting the investigation of anti-inflammatory agents. Candesartan, another ARB, has been shown to reduce cardiac fibrosis by lowering levels of inflammatory markers and collagen synthesis fragments [19]. Additionally, the antioxidant N-acetylcysteine (NAC) exhibits antifibrotic effects by decreasing the production of reactive oxygen species (ROS), thereby improving both systolic and diastolic cardiac functions [20] (Figure 1).

Recent studies have also highlighted the significant role of microRNAs in the regulation of cardiac fibrosis. For instance, miR-29 inhibits collagen synthesis and is considered a negative regulator of fibrosis [21]. Conversely, miR-21 promotes the transformation of quiescent fibroblasts into myofibroblasts, thereby facilitating fibrotic development [22]. Consequently, molecular-targeted therapies aimed at inhibiting miR-21 or activating miR-29 have demonstrated potential in animal models for slowing the progression of fibrosis, although their clinical application remains to be fully validated [23]. Furthermore, regenerative medicine and tissue engineering strategies offer new hope for the treatment of cardiac fibrosis by potentially restoring impaired myocardial function through stem cell transplantation and tissue repair. With the advent of personalized medicine, individualized treatment plans based on patients’ genomic and proteomic profiles are expected to further optimize therapeutic strategies and improve long-term outcomes (Figure 1).

Clinically, cardiac magnetic resonance imaging, particularly with late gadolinium enhancement, enables noninvasive assessment of the distribution and extent of cardiac fibrosis [24]. In addition, serum biomarkers such as type I collagen synthesis fragments, type III collagen synthesis fragments, and matrix metalloproteinases (MMPs) provide dynamic insights into fibrotic progression [25]. These noninvasive diagnostic modalities have greatly enhanced the early detection and precise monitoring of cardiac fibrosis (Figure 1).

## 3. Irisin Is a Key Factor in Cardioprotection

**Figure 2:**
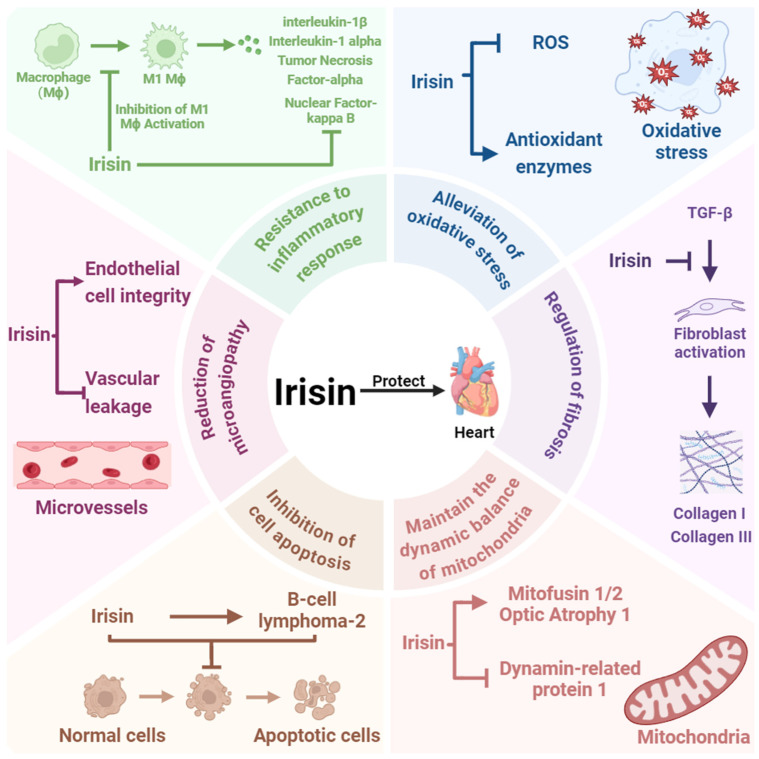
Mechanisms Underlying Irisin-Mediated Cardioprotection. This figure outlines how Irisin exerts cardioprotective effects by inhibiting inflammation, alleviating oxidative stress, maintaining mitochondrial dynamics, suppressing apoptosis, and mitigating microvascular damage. Moreover, Irisin protects cardiac function by reducing fibrosis through the inhibition of the TGF-β signaling pathway. This figure was created in BioRender. Wang, Z. (2025) https://BioRender.com/x59p404, accessed on 7 April 2025.

Irisin was first identified by Boström et al. in 2012 as a cleavage product of FNDC5, generated via PGC-1α activation [26]. Initially, it was believed to increase energy expenditure by inducing the browning of white adipose tissue, which has led to its extensive investigation in obesity research [26]. However, accumulating evidence now suggests that Irisin also plays a crucial role in cardioprotection and holds promise as a predictive biomarker for cardiovascular health. Studies have demonstrated that Irisin can inhibit cardiomyocyte apoptosis by modulating the expression of the pro-apoptotic protein B-cell lymphoma-2 associated X protein [27]. Simultaneously, Irisin increases the expression of antioxidant enzymes (e.g., superoxide dismutase), leading to reduced ROS generation and, consequently, a significant decrease in oxidative damage in cardiomyocytes [28]. These mechanisms are crucial for preventing cardiomyocyte death and dysfunction associated with CVD. Additionally, Irisin promotes mitochondrial fusion by upregulating the expression of mitochondrial fusion proteins, such as mitofusin 1, mitofusin 2, and optic atrophy 1, and inhibits mitochondrial fission by suppressing the activity of the fission protein dynamin-related protein 1. This regulation maintains mitochondrial dynamics and structural integrity, thereby protecting against oxidative stress-induced mitochondrial damage and preserving normal cardiomyocyte function [29,30]. Inflammation is another critical factor in the onset and progression of CVD. Irisin contributes to cardioprotection by inhibiting the activation of the nuclear factor-κB signaling pathway, thereby alleviating chronic inflammatory injury to the myocardium [31]. Moreover, Irisin influences macrophage polarization by suppressing M1 macrophage activation, which in turn reduces the release of proinflammatory cytokines such as tumor necrosis factor-α, interleukin-6, and interleukin-1β (IL-1β) [32]. Fibrosis, a key manifestation of tissue injury in cardiac diseases, is also modulated by Irisin. By regulating macrophage activation, Irisin indirectly affects fibroblast activation. It has been observed that proinflammatory cytokines, such as IL-1β released from macrophages, can stimulate fibroblast proliferation [33,34]. Irisin can inhibit this interaction, thereby slowing the progression of fibrosis [32]. Additionally, Irisin reduces TGF-β-induced fibroblast activation and downregulates the expression of extracellular matrix components such as collagen type I and collagen type III, significantly impeding the progression of fibrosis [35,36]. In FNDC5 gene knockout mouse models, a marked exacerbation of fibrosis has been observed, further underscoring Irisin’s pivotal role in antifibrotic processes [37]. Moreover, Irisin aids in maintaining endothelial cell integrity, reducing vascular leakage, and preserving normal vascular tone, which helps minimize the impact of microvascular damage on the heart, further contributing to cardiac protection [38]. In summary, the cardioprotective mechanisms of Irisin encompass the inhibition of cardiomyocyte apoptosis, reduction in oxidative stress, regulation of mitochondrial function, anti-inflammatory actions, and antifibrotic effects. Irisin thus represents a promising therapeutic candidate for future CVD treatments and opens new avenues for the maintenance of cardiac health (Figure 2).

## 4. The Mechanism by Which Irisin Inhibits Cardiac Fibrosis via the TGF-β/Smad Signaling Pathway

**Figure 3:**
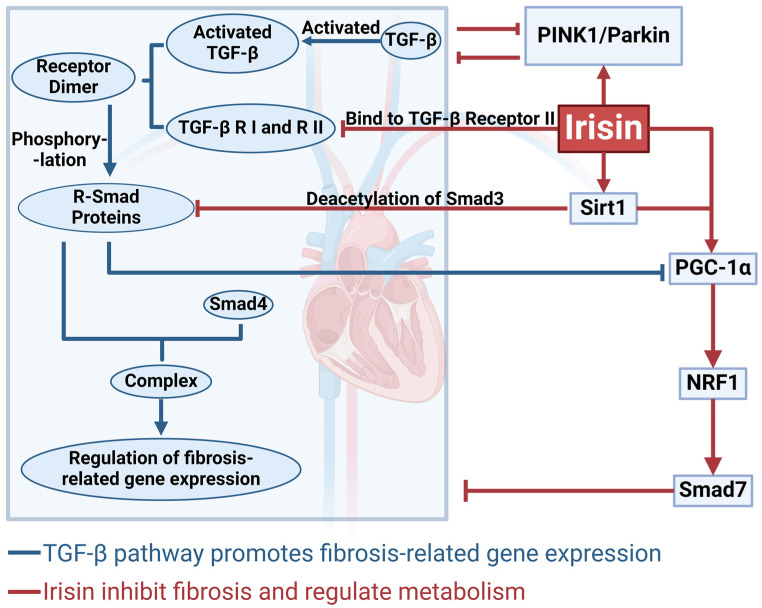
Comprehensive mechanism by which Irisin influences fibrosis Via Sirtuin 1 (Sirt1), peroxisome proliferator-activated receptor gamma coactivator 1-alpha (PGC-1α)/nuclear respiratory factor 1 (NRF-1), and related pathways. This figure summarizes how Irisin significantly reduces the expression of fibrosis-related genes by directly inhibiting TGF-β activation and blocking Smad signaling through receptor binding. Additionally, Irisin activates Sirt1, which mediates the deacetylation of Smad3, further suppressing Smad complex formation. By regulating the PGC-1α/NRF-1 pathway, Irisin helps maintain mitochondrial homeostasis, while enhancing mitophagy through the PTEN-induced kinase 1 (PINK1)/Parkin pathway to clear damaged mitochondria and improve intracellular equilibrium. Collectively, these actions inhibit fibrotic progression, mitigate tissue damage, and offer new insights for the treatment of cardiac fibrosis. This figure was created in BioRender. Wang, Z. (2025) https://BioRender.com/s24j371, accessed on 7 April 2025.

TGF-β acts as the “switch” for fibrosis, existing in a latent state within the extracellular matrix and requiring local proteolysis or mechanical signals for activation [39]. Once activated, TGF-β binds to type I and type II TGF-β receptors on the cell membrane to form receptor dimers. Subsequently, TGF-β receptor I is activated by receptor II, and the signal is transmitted to receptor-regulated Smad (R-Smad) proteins (e.g., Smad2 and Smad3), which become phosphorylated. The phosphorylated R-Smads then complex with Smad4 and translocate into the nucleus, where they regulate the expression of fibrosis-related genes [40]. Studies have found that Irisin can bind to TGF-β receptor II, thereby blocking TGF-β1/Smad signaling and reducing both fibroblast activation and the expression of fibrosis-associated genes [41]. Moreover, Irisin upregulates the expression of Sirt1, further inhibiting fibrosis [42]. Sirt1 is a nicotinamide adenine dinucleotide (NAD^+^)-dependent histone deacetylase that deacetylates Smad3, inhibiting its transcriptional activity and thus attenuating TGF-β1–mediated fibrotic progression [43]. In addition, Sirt1 can enhance the expression of PGC-1α Via deacetylation [44]. Studies have shown that specific deletion of Sirt1 exacerbates fibrotic effects [45]. Furthermore, Sirt1 activates antioxidant and anti-apoptotic pathways, such as those mediated by Forkhead box O and the tumor suppressor protein p53, thereby protecting the myocardium from TGF-β1–induced oxidative stress and apoptosis [46]. Sirt1 also promotes angiogenesis and myocardial repair by activating vascular endothelial growth factor [47]. Thus, the Irisin–Sirt1–TGF-β regulatory axis plays a crucial protective role in cardiac fibrosis (Figure 3).

PGC-1α is a key regulator of mitochondrial biogenesis. Studies have demonstrated that TGF-β1, Via Smad3 binding to the promoters of FNDC5 and PGC-1α, suppresses their mRNA and protein expression, thereby impairing mitochondrial function [48]. TGF-β1 can also inhibit mitochondrial autophagy by suppressing the microtubule-associated protein 1A/1B-light chain 3 (LC3) and the PTEN-induced kinase 1 (PINK1)/Parkin pathways [49]. Consequently, the TGF-β1/Smad signaling pathway further exacerbates fibrosis by compromising mitochondrial function. Exogenous administration of Irisin has been shown to upregulate PGC-1α expression [50]. PGC-1α promotes mitochondrial autophagy by regulating lysosomal and LC3 pathways and activates NRF-1 to enhance mitochondrial transcription. Moreover, NRF-1 can bind to the promoter of FUN14 domain containing 1 (Fundc1), increasing the expression of the mitochondrial autophagy receptor Fundc1 and interacting with LC3 to further promote mitophagy [51]. These processes are essential for preventing cardiac fibrosis that results from mitochondrial dysfunction. Additionally, there is cross-talk between the NRF-1 and TGF-β1/Smad signaling pathways. NRF-1 can bind to the Smad7 promoter, enhancing its expression; since Smad7 serves as a negative feedback inhibitor of TGF-β1 signaling, this interaction helps prevent excessive fibrosis [52]. Furthermore, Irisin interacts with the PINK1/Parkin pathway, further mitigating TGF-β1–induced mitochondrial damage [53]. Thus, by activating the PGC-1α/NRF-1 signaling pathway, Irisin counteracts the deleterious effects of TGF-β1 on mitochondrial function, thereby reducing cardiac fibrosis. Interestingly, the regulatory role of Irisin in cardiac fibrosis is complex, with its effects varying dramatically depending on the dosage. Mitogen-Activated Protein Kinase (MAPK) plays a central role in the transition of fibroblasts to myofibroblasts and is considered a potential target for anti-fibrotic therapy [54]. Inhibition of the MAPK pathway has been shown to effectively reduce Ang II–induced cardiac remodeling in mice and improve functional impairments [55]. Moreover, several cardiac fibrosis–related conditions, including heart failure and myocardial infarction, can be alleviated by suppressing MAPK signaling [56,57]. Research indicates that low-dose Irisin treatment alleviates cardiac fibrosis by increasing Smad7 expression and inhibiting the phosphorylation of Smad2 and Smad3, thereby suppressing the endothelial-to-mesenchymal transition Via the TGF-β/Smad pathway; high-dose Irisin may excessively activate the MAPK pathway, disrupt the balance of MMP, and ultimately exacerbate fibrosis [58]. Therefore, optimizing Irisin dosage will be critical for achieving the best therapeutic outcomes in future treatment strategies. In summary, Irisin exhibits significant anti-fibrotic effects in the heart by modulating multiple signaling pathways—particularly the TGF-β/Smad, Sirt1, and PGC-1α/NRF-1 pathways—which work synergistically to form a complex signaling network. This network offers promising therapeutic targets for the prevention and treatment of cardiac fibrosis (Figure 3).

## 5. Exercise Promotes Irisin Release to Regulate Cardiac Fibrosis

**Figure 4:**
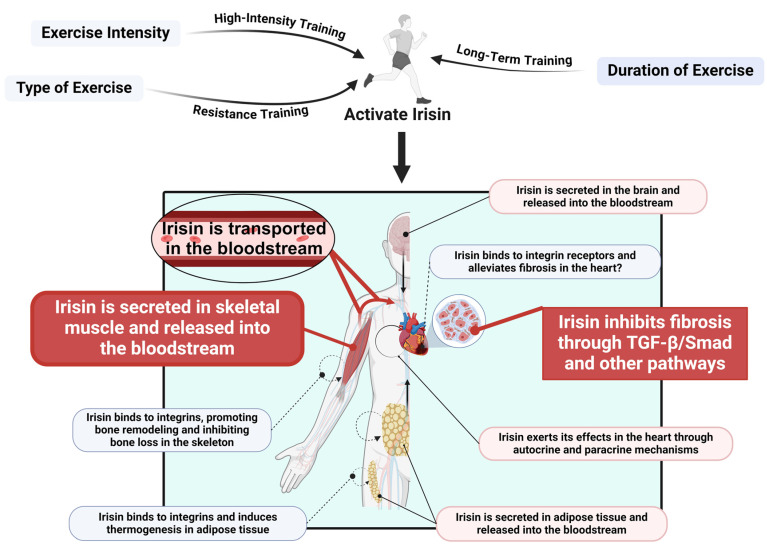
Exercise-Induced Irisin Release and Its Role in Cardiac Fibrosis. Exercise (high-intensity, resistance, and long-term training) activates Irisin secretion from skeletal muscle. Irisin enters the bloodstream and exerts systemic effects. In the heart, it binds to integrin receptors, modulating the TGF-β/Smad signaling pathway to inhibit fibrosis. Additionally, Irisin functions through autocrine and paracrine mechanisms. It also influences thermogenesis in adipose tissue and bone remodeling, highlighting its multifunctional role. This figure was created in BioRender. Wang, Z. (2025) https://BioRender.com/o81x119, accessed on 7 April 2025.

Exercise has been shown to effectively ameliorate cardiac fibrosis. Studies indicate that after 5 weeks of exercise training, significant alterations occur in fibroblast growth factor 21 and the TGF-β1–Smad2/3–MMP2/9 signaling pathway in fibroblasts, resulting in a reduction in cardiac collagen fiber synthesis and a subsequent alleviation of fibrosis [59]. In fact, Irisin is an exercise-induced hormone, also known as a myokine, because its release is triggered by the upregulation of the FNDC5 gene in skeletal muscle [60]. Moreover, Irisin is produced by a variety of tissues; it is not only secreted by skeletal muscle but also released by adipose tissue, the heart, the brain, and other organs [61]. Once secreted, Irisin is transported Via the bloodstream to distant tissues where it exerts its physiological functions. In addition to its endocrine role, Irisin may also act locally Via paracrine mechanisms. For instance, in models of doxorubicin-induced cardiotoxicity and perivascular fibrosis, local diffusion of Irisin—secreted by cardiomyocytes within the cardiac microenvironment—significantly mitigated the progression of fibrosis by exerting protective effects on endothelial cells through paracrine signaling [31]. Other studies have suggested that Irisin may also function through an autocrine mechanism, wherein cells secrete Irisin and respond to it Via their own receptors [62]. The potential roles of these paracrine and autocrine pathways in local myocardial regulation are emerging as a research hotspot. However, these studies are still in their early stages, and further exploration and validation are required. Notably, integrins, which are considered potential receptors for Irisin, may play an important role in the improvement of cardiac fibrosis. Research has demonstrated that Irisin can bind to integrins, inducing thermogenic mechanisms in adipose tissue, promoting bone remodeling, and inhibiting bone loss in the skeletal system [63]. These findings support the concept that Irisin exerts its biological effects Via integrin receptors. Nonetheless, the precise mechanisms by which Irisin functions in cardiac tissue remain to be elucidated. Future investigations employing receptor screening techniques may provide deeper insights into the targeting mechanisms of Irisin in the heart, thereby clarifying its specific role in alleviating fibrosis (Figure 4).

The effects of exercise on Irisin secretion are complex and are jointly regulated by exercise intensity, type, and duration. Different exercise intensities significantly influence Irisin secretion levels. Studies have shown that under similar energy expenditure conditions, high-intensity exercise (80% VO_2_max) leads to significantly higher Irisin concentrations at 6 and 19 h post-exercise compared to low-intensity exercise (40% VO_2_max) [64]. Further research has indicated that resistance training is more effective than endurance training, or a combination of endurance and resistance training, in promoting Irisin secretion [65]. This finding is consistent with other reports, such as those by Huh et al. [66], who observed that circulating Irisin levels following resistance training were higher than those seen after high-intensity interval training or moderate-intensity continuous exercise. The duration of exercise also markedly affects the persistence of Irisin secretion. While acute bouts of high-intensity exercise can rapidly elevate Irisin levels, long-term regular training (e.g., sustained high-intensity exercise) tends to increase the baseline concentration of Irisin. For example, a study involving healthy young subjects (seven males and five females) demonstrated that during a 90 min treadmill aerobic exercise at 60% VO_2_max, Irisin levels significantly increased at 54 min into the exercise session, but returned to baseline immediately after the 90 min session and following a 20 min recovery period [67]. Another study reported that sedentary individuals had a baseline plasma Irisin concentration of approximately 3.6 ng/mL, which significantly increased to 4.3 ng/mL after 12 weeks of high-intensity aerobic training [68]. In addition to exercise parameters, environmental factors also influence Irisin secretion. One study found that aerobic exercise performed in a 0 °C environment significantly increased Irisin levels, whereas no significant changes were observed before and after exercise in 12 °C and 24 °C settings [69]. Given the multifaceted protective role of Irisin in cardiac fibrosis, inducing its release through exercise emerges as a potential non-pharmacological therapeutic strategy. Both in vivo and in vitro studies have demonstrated that exercise-induced Irisin expression upregulates Sirt1 activity and suppresses the TGF-β1/Smad signaling pathway, thereby mitigating the extent of cardiac fibrosis [42]. In summary, exercise stimulates the secretion of Irisin, which in turn alleviates the progression of cardiac fibrosis through endocrine, paracrine, and possibly autocrine mechanisms. Future research should further explore the targeted actions of Irisin in cardiac tissue and elucidate its specific molecular mechanisms to provide a robust scientific basis for non-pharmacological interventions in cardiac fibrosis (Figure 4).

## 6. Conclusions

Irisin, as an exercise-induced cardioprotective factor, exhibits broad potential in antifibrotic, anti-inflammatory, and mitochondrial function regulatory effects. Its multi-target regulatory mechanisms offer new therapeutic directions for cardiac fibrosis. In addition, Irisin exhibits protective effects in various tissues, suggesting its potential application beyond cardiac fibrosis to other fibrosis-related diseases, such as liver and pulmonary fibrosis. Despite the progress in understanding Irisin’s role in cardiac function regulation, several key questions remain unanswered. For instance, the precise mechanisms of Irisin under different pathological conditions and its interactions with other cardiovascular regulatory factors require further investigation. Optimizing the dosage and administration of Irisin may lead to more personalized and effective therapeutic strategies for CVD prevention and treatment. Additionally, whether exogenous Irisin administration can mimic the cascade effects induced by exercise warrants further investigation, as it may offer an alternative biotherapeutic strategy for patients with limited mobility. In summary, exercise-induced Irisin release provides novel insights for the prevention and treatment of cardiovascular diseases, fostering a transition from empirical exercise intervention recommendations to a mechanism-oriented precision medicine approach, and opening new avenues for exploring the molecular mechanisms underlying exercise-mediated cardiac health.
